# Unexpected High Sensory Blockade during Continuous Spinal Anesthesiology (CSA) in an Elderly Patient

**DOI:** 10.1155/2012/648921

**Published:** 2012-08-13

**Authors:** R. Ketelaars, A. P. Wolff

**Affiliations:** Department of Anaesthesia, Radboud University Nijmegen Medical Centre, P.O. Box 9101, 6500 HB Nijmegen, The Netherlands

## Abstract

A 98-year-old woman presented for a hemiarthroplasty of the left hip. Because of her age and cardiac and pulmonary co-existing diseases we decided to provide adequate regional anesthesia by continuous spinal anesthesia. Fragmented doses of isobaric bupivacaine 0.5% were administered through a system consisting of a spinal catheter connected to an antimicrobial filter. After an uneventful surgical procedure, prior to removal of the catheter, this system was flushed with 10 mL of normal saline in order to try to prevent post-dural-puncture headache. After arrival at the postanesthesia care unit and fifteen minutes after removal of the catheter the patient suffered an unexpected high thoracic sensory blockade and hypotension requiring treatment. The continuous spinal anesthesia technique can be used in selected cases to be able to administer local anesthetic agents in a slow and controlled manner to reach the desired effect. The risk of post-dural-puncture headache using this technique in elderly patients is very low and therefore precludes the need to try to prevent it. We have described a potentially dangerous complication of flushing a bupivacaine-filled system into the spinal canal of an elderly patient resulting in an undesirable high sensory blockade.

## 1. Introduction

Continuous spinal anesthesia (CSA) is an accepted technique of achieving regional anesthesia for surgery below the waist in elderly patients [1, 2]. Its advantages are the superior control by the physician on the type and dosage of drugs administered intrathecally. Consequently the level of sensory blockade can be controlled very precisely leading to minimal respiratory, hemodynamic and mental implications [3].

The height of a sensory block achieved with a single-shot spinal anesthesia (SSA) depends on various patient factors including age [4]. Advanced age also influences cardiovascular instability following spinal anesthesia, CSA may therefore be preferred over SSA for elderly patients [5]. Moreover the extent and intensity of the intrathecal blockade induced with local anesthetic agents influences cardiovascular instability. Concomitant conditions like cardiomyopathy or aortic valve stenosis may increase this risk of hemodynamic instability and cardiovascular complications further, strengthening the indication for CSA.

The downside of CSA over SSA is its higher rate of complications such as infection, epidural hematomas, post-dural-puncture headache (PDPH), and cauda equina syndrome [6–9]. 

This report is, as far as we know, the first one describing unintentional and unexpected high sensory blockade following CSA. We do not believe this to be an uncommon or even unrecognized complication but it nevertheless appears to be an underreported one.

## 2. Case Presentation

A 98-year-old woman suffered a left-sided femoral neck fracture caused by a fall and presented for a hip hemiarthroplasty. Her medical record shows paroxysmal atrial fibrillation, hypertension, recurrent complaints attributed to cardiac decompensation, a cardiac murmur and hypoxemic chronic obstructive pulmonary disease (COPD), global initiative for chronic obstructive lung disease (GOLD) classification stage II (moderate severity), for which she received supplemental oxygen therapy at home. Her symptoms of dyspnea have remained constant over the last few weeks. She had contracted pulmonary tuberculosis in the past and had recently been treated with an antibiotic and prednisolone because of recurrent pneumonia and COPD exacerbation. Over the last few years the number of falls she sustained had increased.

Additional medication consisted of a loop diuretic, calcium channel inhibitor, nebulized combination of a *β*2-agonist and an anticholinergic agent, and acetaminophen. She took no antiplatelet drugs or vitamin-K antagonists.

On physical examination she had a respiratory frequency of 17 per minute, peripheral oxygen saturation (SpO_2_) 92% with nasal administration of O_2_ at two liters per minute. Blood pressure was 144/72 mmHg and heart rate 81 beats per minute (bpm). A cardiac murmur was heard over the chest. Electrocardiography showed a normal sinus rhythm.

The transthoracic echocardiogram revealed a moderate aortic valve stenosis and a hypertrophied left ventricle with a good systolic function.

Because of the patients advanced age, aortic valve stenosis and the kind of surgery required, we formulated a plan to pursue a hemodynamic situation within normal limits for this patient with minimal impact on pulmonary and cerebral function. We decided to provide adequate regional anesthesia by CSA.

We undertook this procedure starting an hour before surgery at the postanesthesia care unit (PACU) with monitoring of pulse, ECG, and blood pressure. In a complete sterile manner with the patient in a sitting position we introduced an 18-gauge Tuohy needle (Perican; B. Braun Melsungen AG, Melsungen, Germany) in the midline at the level of the spinous process of the third and fourth lumbar vertebrae. After we punctured the duramater and observed a free flow of spinous fluid, we introduced a 20 gauge, 104 cm catheter (Perifix Softtip; B.Braun), and five centimeters intrathecally. This catheter was connected to an antimicrobial filter (Perifix; B.Braun), flushed with normal saline (NaCl 0.9%), and fixed to the patients back with adhesive film and tape. This procedure using these materials describes the standard operating procedure to introduce an intrathecal catheter for CSA in our institution.

Approximately 45 minutes before surgery we administered 0.5 mL of a 0.5% isobaric bupivacaine solution (2.5 mg; AstraZeneca, London, United Kingdom). Fifteen minutes later we administered another 0.25 mL (1.25 mg) of this solution. To assess the extent of sensory blockade a refrigerated metal “hammer” was used. The patient was able to adequately report cold sensation while touching the skin of the thorax, abdomen, and upper legs on both sides of the body, moving in a craniocaudal direction. Five minutes after having administered the second dose of the local anesthetic agent we tested sensory blockade and determined it to be symmetrically at the level of the eleventh thoracic dermatome and below. After this we introduced a urinary catheter and placed the patient in the right dependent position and surgery commenced.

An hour after the second dose of bupivacaine we administered a third dose of 0.5 mL (2.5 mg) and another hour later a fourth dose of 0.5 mL. Upon completion of the surgery and 50 minutes after the last dose of bupivacaine, we removed the spinal catheter. Immediately prior to removing the catheter we injected 10 mL of normal saline to reduce the risk of developing PDPH and the patient was transported to the PACU.

During surgery all monitored physiological variables stayed within normal values. We administered 500 mL of a colloidal solution and 250 mL of normal saline. The course of surgery was as planned. Blood loss was 500 milliliters.

Fifteen minutes after removal of the catheter her blood pressure decreased to a minimum of 75/35 mmHg with a heart rate of 80 bpm. After administration of up to a total of 350 *μ*g of phenylephrine, her pulse slowed to 60 bpm and blood pressure improved. Examination revealed an unexpected symmetric sensory blockade at the third thoracic dermatome and below. She reported no pain and was unable to move her legs and feet.

Besides frequent hemodynamic monitoring, sensory blockade was monitored every 15 minutes. We considered requesting a magnetic resonance imaging scan to rule out an epidural hematoma being a deleterious side effect of introducing or removing neuraxial catheters. Fortunately 130 minutes after the first occurrence of high sensory blockade and hypotension, sensory blockade finally regressed to a level below the sixth thoracic dermatome. Thereafter motor and sensory blockade regressed swiftly.

Pain after regression of the blockade was successfully treated with fragmented doses of intravenous morphine.

The patient was discharged on the fifth day after surgery after an otherwise uneventful hospital stay. She never complained about headaches.

## 3. Discussion

The CSA technique can be used to titrate local anesthetic agents to accomplish an adequate height of sensory blockade for patients having surgery. The wide spread of local anesthetic agents when using a CSA technique can in many cases occur after start of titration of the doses, if they are administered too fast or in higher doses than necessary. This adverse effect is thought to be known by most anesthesiologists. While there are reports of intrathecal catheters resulting in abnormal spread or inadequate sensory blocks, to date there are no reports in medical literature of the potentially dangerous complication we have described [10].

We hypothesized that the 10 mL flush of normal saline prior to the removal of the intrathecal catheter caused the late and high level of the unintended intrathecal anesthesia. The system we used consisted of a 104 cm catheter connected with a connector to an antimicrobial filter, closed with a cap. This system contains a total volume of 0.8 mL. In the worst case scenario this system would contain undiluted bupivacaine 0.5% and thus a dose of 4 mg. By flushing the system with normal saline we diluted and flushed most of the bupivacaine into the cerebrospinal fluid (CSF). We hypothesized that the resulting effect resembled a technique called barbotage in which during the performance of a SSA a local anesthetic agent is injected while intermittently CSF is aspirated and reinjected to dilute the local anesthetic and to induce a certain current in the CSF. The rationale of the barbotage technique is to increase sensory block height using a relatively low dose of local anesthetic drugs, however evidence is lacking [4, 11].

Elderly patients may have a lower lumbosacral cerebrospinal fluid (CSF) volume with lower CSF pressures [12]. The combination of 4 mg bupivacaine and its dilution with 10 mL normal saline into a relatively small volume of CSF might also have contributed to the unexpected large extent of intrathecal anesthesia up to the 3rd thoracic dermatome.

When using the CSA technique, the risk of PDPH is slightly higher, but probably still very low. By injecting normal saline, we intended to reduce the risk of PDPH, despite the incidence of this occurring after an SSA decreases with age [13]. In addition there appears to be little evidence, if any, for a single intrathecal injection of normal saline before removing a catheter to prevent the occurrence of post-dural-puncture headache [9, 14, 15].

In conclusion we recommend that regional anesthesia by CSA be the technique of choice in the elderly high-risk patient requiring hip surgery. In this case the procedure went well until we flushed the intrathecal catheter with 10 mL saline before catheter removal. This probably caused an unintended and undesirable high sensory blockade because the intrathecal system contained enough bupivacaine to block the spinal afferent and efferent nerves up to a high thoracic level in an elderly patient.

This case demonstrates that the injection of normal saline through the intrathecal catheter before removal in order to reduce the chance on PDPH in elderly patients is debatable and not without risk. Anesthesiologists may be aware of this potential event, but as far as we know an incident like this has not been reported in literature previously.
